# Progression of Pathogenic Events in Cynomolgus Macaques Infected with Variola Virus

**DOI:** 10.1371/journal.pone.0024832

**Published:** 2011-10-06

**Authors:** Victoria Wahl-Jensen, Jennifer A. Cann, Kathleen H. Rubins, John W. Huggins, Robert W. Fisher, Anthony J. Johnson, Fabian de Kok-Mercado, Thomas Larsen, Jo Lynne Raymond, Lisa E. Hensley, Peter B. Jahrling

**Affiliations:** 1 Integrated Research Facility at Fort Detrick, National Institute of Allergy and Infectious Diseases, National Institutes of Health, Frederick, Maryland, United States of America; 2 Whitehead Institute for Biomedical Research, Massachusetts Institute of Technology, Cambridge, Massachusetts, United States of America; 3 United States Army Medical Research Institute of Infectious Diseases, Fort Detrick, Maryland, United States of America; Tulane School of Public Health and Tropical Medicine, United States of America

## Abstract

Smallpox, caused by variola virus (VARV), is a devastating human disease that affected millions worldwide until the virus was eradicated in the 1970 s. Subsequent cessation of vaccination has resulted in an immunologically naive human population that would be at risk should VARV be used as an agent of bioterrorism. The development of antivirals and improved vaccines to counter this threat would be facilitated by the development of animal models using authentic VARV. Towards this end, cynomolgus macaques were identified as adequate hosts for VARV, developing ordinary or hemorrhagic smallpox in a dose-dependent fashion. To further refine this model, we performed a serial sampling study on macaques exposed to doses of VARV strain Harper calibrated to induce ordinary or hemorrhagic disease. Several key differences were noted between these models. In the ordinary smallpox model, lymphoid and myeloid hyperplasias were consistently found whereas lymphocytolysis and hematopoietic necrosis developed in hemorrhagic smallpox. Viral antigen accumulation, as assessed immunohistochemically, was mild and transient in the ordinary smallpox model. In contrast, in the hemorrhagic model antigen distribution was widespread and included tissues and cells not involved in the ordinary model. Hemorrhagic smallpox developed only in the presence of secondary bacterial infections – an observation also commonly noted in historical reports of human smallpox. Together, our results support the macaque model as an excellent surrogate for human smallpox in terms of disease onset, acute disease course, and gross and histopathological lesions.

## Introduction

Smallpox is widely regarded as a scourge of epic proportions whose history dates back to ca.1350 BCE when smallpox spread from the Egyptians to the Hittites in Syria [1]. It has been recorded throughout human history with the last naturally occurring cases being reported in 1975 in the Indian subcontinent and in 1977 in the Horn of Africa [1]. The eradication of human smallpox, conducted under the auspices of the World Health Organization (WHO), involved a worldwide campaign of ring vaccination, and vigorous tracking of individual smallpox cases [2], [3], [4], [5]. The success of the campaign was due, in part, to the lack of animal reservoirs that could reintroduce the virus into the human population. Following eradication, the remaining stocks of VARV were either consolidated into two maximum-containment facilities designated as the WHO Collaborating Centers for Smallpox and Other Poxvirus Infections, or destroyed [6]. Beginning in 1980, the vaccination program for smallpox was dismantled, resulting in the world's population now being largely susceptible to this disease. Unfortunately, following eradication, the USSR developed VARV as a strategic bioweapon [7], [8], and concerns remain that VARV has the potential to be used as an agent of bioterrorism. For this reason, improved countermeasures against smallpox including new vaccines and antiviral drugs are urgently needed.

Development of countermeasures for biothreat agents requires demonstration of efficacy in an animal model infected with the authentic agent resulting in a progression of events recapitulating the human disease process [9]. In humans, the clinical manifestations of naturally acquired smallpox begin after an incubation period of 7–17 days following exposure. The initial symptoms of disease are indistinguishable from many other virus infections and may include backache, headache, vomiting, prostration and fever that can spike upwards of 39°C. The febrile phase may continue for 3–4 days before the appearance of a centrifugally distributed, systemic rash. The rash progresses from a macular to papular stage, after which vesicles evolve on day 4 and pustules on day 7 post fever onset. The classical form of smallpox, caused by variola major, has been divided into five clinical types by the WHO [10]: 1) ordinary smallpox, occurred in 90% of cases (case-fatality rate correlated with the severity of rash and ranged from 10–75%); 2) modified smallpox (5% of the cases) was less severe in clinical presentation (case-fatality rate ∼10%) and frequently occurred in previously vaccinated individuals; 3) Variola sine eruption presented as a fever without rash and serological evidence was required to confirm infection was due to VARV; 4) flat smallpox occurred in 5% of cases and was distinguished by a more slowly developing rash and generalized infection (case-fatality rate ≥80%); and 5) hemorrhagic smallpox (1% of cases), characterized by hemorrhages into the skin and mucous membranes and uniform lethality [10].

Even though the clinical presentation and pathology of human smallpox have been previously documented [11], [12], [13], [14], much of smallpox pathogenesis remains to be characterized. The development of an animal model to recapitulate this disease progression has been hindered by the host range of natural VARV which is restricted to humans. Nevertheless, initial studies to explore the potential of VARV infection in cynomolgus macaques yielded promising results, with animals developing fulminant and lethal infection with end-stage lesions reminiscent of human disease. Studies by Jahrling *et al* identified a dose-dependent disease course in which high dose inoculation resulted in hemorrhagic diathesis and 10-fold lower doses produced ordinary smallpox [15]. By their nature, those preliminary studies did not include temporal pathology analysis. Thus, the current serial pathogenesis study was performed in cynomolgus macaques that were inoculated intravenously (iv) with either 10^8^ plaque forming units (pfu) or 10^9^ pfu doses of VARV designed to produce ordinary-like or hemorrhagic-like smallpox, respectively. Animals were monitored for clinical signs of disease including lesion counts, temperature, body weight, serum chemistries, and hematology analysis. Additionally, samples acquired at necropsy were evaluated histologically, immunohistochemically, and ultrastructurally. Quantitative real-time polymerase chain reactions (qrt-PCRs) and enzyme-linked immunosorbent assays (ELISAs) were used to measure viremia and immune mediators, respectively. These studies significantly advance our understanding of VARV pathogenesis in primates and increase confidence in the VARV cynomolgus monkey model for facilitating development of antiviral drugs, improved countermeasures, and suggesting potential targets for therapeutic intervention in humans.

## Results

### Comparative Pathology of VARV Infection

#### Gross Necropsy Findings

10^8^ pfu Group.

Eighteen vaccinia virus-naïve cynomolgus macaques (*Macaca fascicularis*) were inoculated intravenously (iv) with 10^8^ pfu VARV Harper strain and necropsied on days 1, 3, 5, 7, 9 and 11 post infection (n = 3/time point). The major postmortem findings are listed in Table 1. Significant lesions first became apparent on day 3 and were limited to peripheral and visceral lymphadenopathy and prominent splenic lymphoid follicles in one animal only. Peripheral lymphadenopathy developed in all animals by day 5, persisted throughout the study, and increased in severity as the disease progressed. Exanthema and enanthema first became apparent on day 5 and consisted of numerous macules and papules on the legs, arms, face, lips, and tongue of all animals. Mean cutaneous lesion counts, in excess of 1,000, peaked between days 7 and 8 and distribution expanded to include the palmar and plantar surfaces, tail, scrotum, and prepuce. Oral mucosal lesions peaked at day 5 and declined to a single umbilicated pustule on the tongue of one animal by day 11. Additional findings included multifocal testicular hemorrhage and splenic congestion.

**Table 1:** Major Gross Pathology Findings – 10^8^ pfu group.

Lesion	Day 1	Day 3	Day 5	Day 7	Day 9	Day 11
Lymphadenopathy	0	1	3	3	3	3
Exanthema	0	0	3	3	3	3
Enanthema	0	0	3	3	1	1
Splenic Congestion	1	1	2	0	1	1
Testicular Hemorrhage	0	0	2	1	0	0

Values indicate the number of animals affected per group of 3.

#### Gross Necropsy Findings

10^9^ pfu Group.

Nine vaccinia virus-naïve cynomolgus macaques (*Macaca fascicularis*) were inoculated intravenously with 10^9^ pfu VARV Harper strain and scheduled for necropsy on days 1, 3, and 4 post infection (n = 3/time point). The time points chosen for this group were based on the accelerated disease course previously observed [15]. All of the day 1 and day 3 animals were euthanized according to schedule. The day 4 animals developed a secondary bacterial infection and were found dead on days 2 (n = 1) and 3 (n = 2). The major postmortem findings are listed in Table 2. In contrast to the 10^8^ pfu group, poxviral papules and pustules were not found, and cutaneous lesions were limited to a petechial rash in 2/3 of the animals euthanized on day 3. One of these animals also had a single focus of colonic mucosal hemorrhage. Among the animals found dead, 2 had multifocal hemorrhages within the urinary bladder mucosa, and of those, 1 had additional hemorrhage in the cecal mucosa and irides. The third animal in this group had focal hemorrhage within the peripancreatic mesentery. Hepatic congestion was present in all of the animals found dead, and splenic congestion was found in two of the animals euthanized on day 3. Peripheral and visceral lymphadenopathy was found in all of the animals euthanized on day 3 and a single animal found dead.

**Table 2:** Major Gross Pathology Findings – 10^9^ pfu group.

Lesion	Day 1 (euthanized)	Day 3 (euthanized)	[Table-fn nt103]Day 4* (Premature deaths)
Petechial Rash	0	2	0
Intestinal Hemorrhage	0	1	1
Other Hemorrhage	0	0	3
Splenic Congestion	0	2	1
Hepatic Congestion	0	0	3
Lymphadenopathy	0	3	1

Values indicate the number of animals affected per group of 3.

*animals scheduled for euthanasia on day 4 succumbed on days 2 or 3.

#### Histopathology

10^8^ pfu Group.

**Figure 1:**
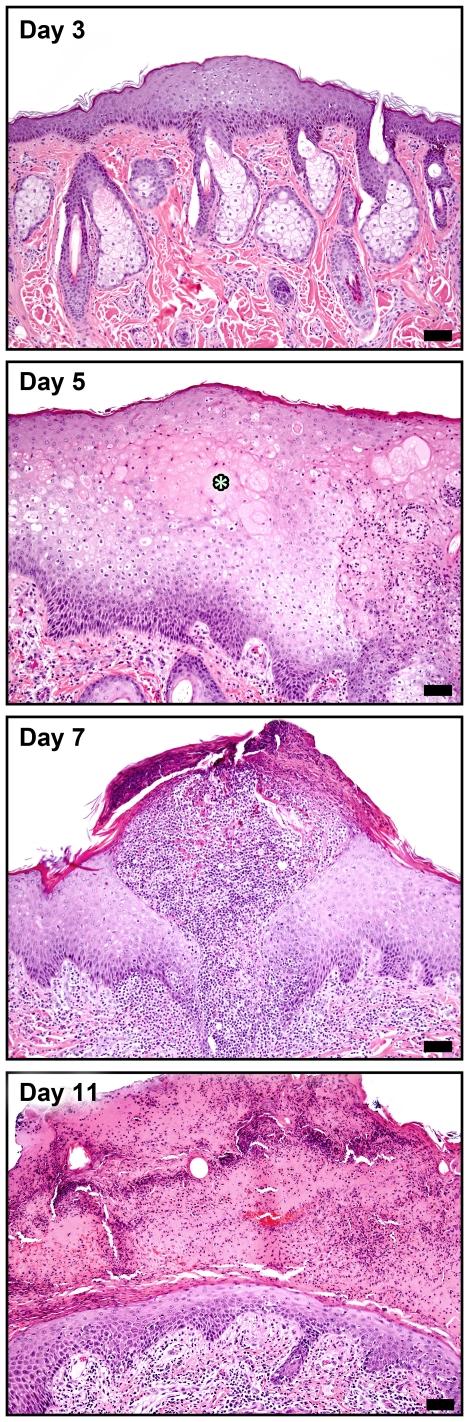
Progression of cutaneous lesions in the 10^8^ pfu group. Day 3: Focal area of mild epidermal hyperplasia and keratinocyte swelling. Day 5: Marked epidermal hyperplasia with ballooning degeneration and early vesicle formation (*). Day 7: Intraepidermal pustule and early crust formation; dermal inflammation is primarily neutrophilic. Day 11: Resolution of epidermal hyperplasia and replacement by large serocellular crust; dermal inflammation is primarily lymphohistiocytic, H&E 10X; mag bars = 50 um.

Epitheliocentric lesions were commonly found in haired skin and oronasal mucosa. Cutaneous lesions became apparent histologically at day 3, and consisted of mild epithelial cell swelling and hyperplasia which progressed to ballooning degeneration and necrosis with typical intraepidermal vesicles and eosinophilic intracytoplasmic inclusion bodies (ICIB) by day 5 (Figure 1). At days 7 and 9, epidermal and follicular pustules were apparent, and by day 11, epidermal crusts and lymphoplasmacytic dermatitis predominated. Mucosal lesions were similar and commonly involved nasal, tonsillar and glossal epithelium. Despite the relative lack of gross oral mucosal lesions at days 9 and 11, histopathologic lesions were readily apparent. Immunohistochemical (IHC) staining for poxviral antigen was commonly found in cutaneous and mucosal epithelial cells surrounding and within the lesions.

**Figure 2:**
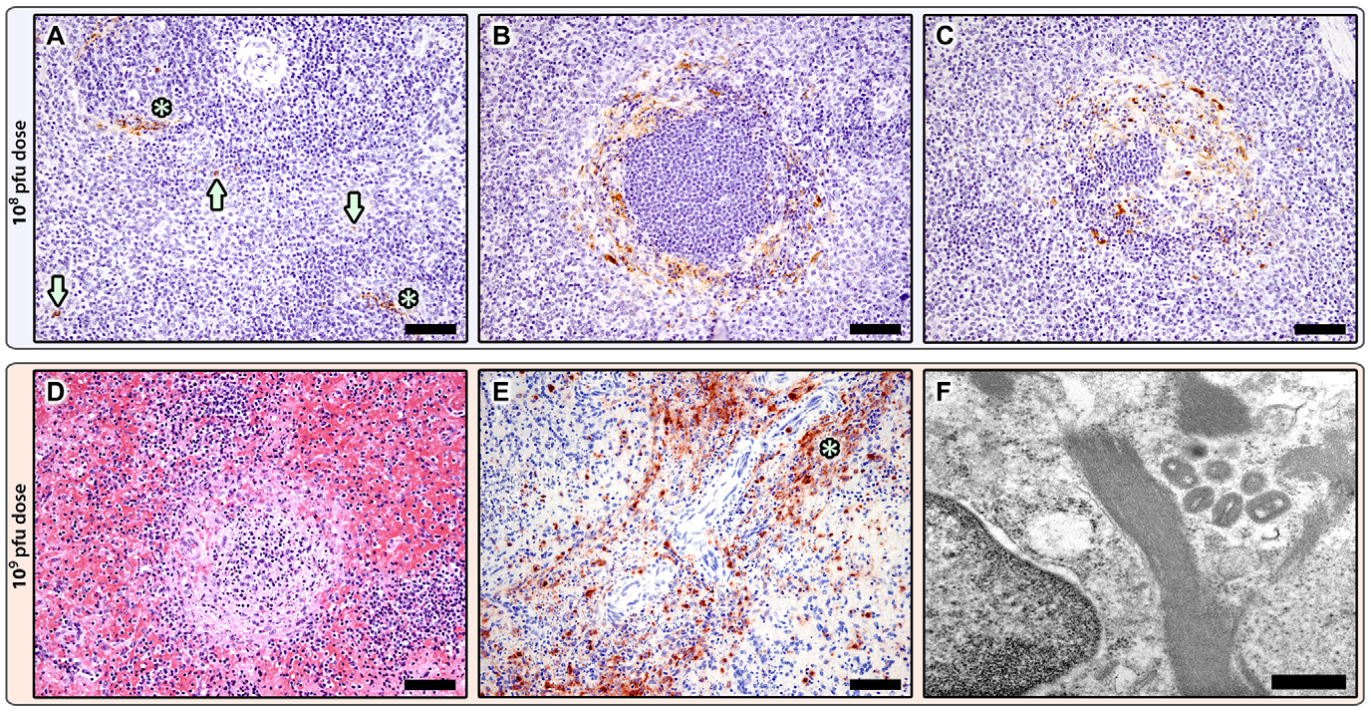
Spleen. A–C: Poxviral immunohistochemistry in the 10^8^ pfu group. A: Day 1, Positive staining (brown) is localized to the marginal zone of primary follicles (*) and scattered individual cells within the sinusoids (arrows). Additional positive staining on Day 3 within the marginal zone of primary follicles (B) and within early germinal centers (C), anti-vaccinia IHC; 20X; mag bars = 50 um. D-F: Splenic changes in the 10^9^ pfu premature death group. D: Diffuse red pulp hemorrhage and congestion, and marked white pulp lymphocytolysis and lymphoid depletion, H&E 20X; mag bar = 50 um. E: Widespread poxviral IHC staining (brown) is localized to the periarteriolar lymphoid sheaths (*), individual cells within the sinusoids (arrows), and follicular marginal zones (not shown), anti-vaccinia IHC; 20X; mag bar = 50 um. F: Ultrastructural appearance of mature virions, fibrin, and a degenerate cell, TEM; 40000X; mag bar = 500 nm.

Splenic changes were common and included mild congestion and red pulp neutrophilia on day 1 and minimal to mild lymphoid hyperplasia on days 5 through 11. The splenic cords and sinusoids were expanded by large numbers of plasma cells and macrophages by days 9 and 11. Immunohistochemistry revealed distinct poxviral antigen staining within the marginal zone of primary follicles, as well as occasional single cells within the sinusoids, on day 1 (Figure 2). On day 3, marginal zone staining persisted and additional staining was found centrally in early germinal centers (Figure 2). Regardless of location, staining was localized to the cytoplasm of large pleomorphic to stellate cells with vesicular nuclei and abundant cytoplasm. No viral antigen staining was found on days 5–11.

**Figure 3:**
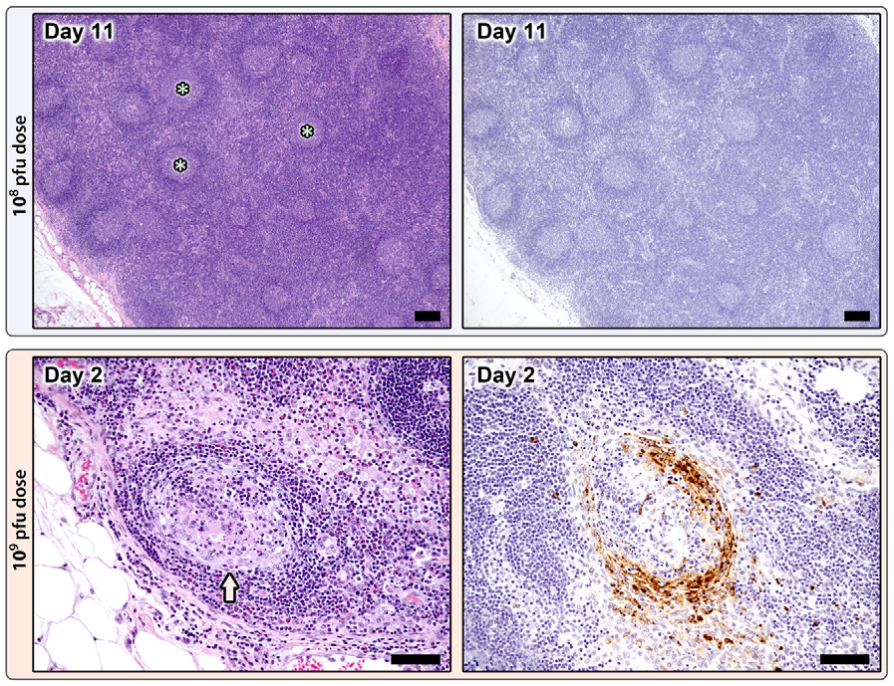
Lymph node changes in the 10^8^ pfu (top) and 10^9^ pfu (bottom) dose groups. 10^8^ pfu dose, Day 11: Marked lymphoid hyperplasia with large numbers of secondary follicles containing well developed germinal centers (*), H&E 5X; mag bar = 100 um. Poxviral immunohistochemistry (right) is negative, anti-vaccinia IHC; 5X; mag bar = 100 um. 10^9^ pfu dose, premature death group, Day 2: Follicular depletion and lymphocytolysis with abundant apoptotic debris, H&E 20X; mag bar = 50 um. Poxviral IHC staining (brown) is localized to the mantle zone and scattered individual cells within the paracortex and sinuses, anti-vaccinia IHC; 20X; mag bar = 50 um.

Mandibular, mediastinal, axillary, inguinal, and mesenteric lymph nodes were examined. Lymphoid hyperplasia became apparent at day 5 and was most developed by day 11 (Figure 3). Marked subcapsular and medullary sinus histiocytosis, and mild hemorrhage, edema, and neutrophilia were present throughout the study. As in the spleen, by days 9 and 11 the medullary cords were expanded by large numbers of plasma cells. No positively stained cells were identified by IHC at any time point (Figure 3).

**Figure 4:**
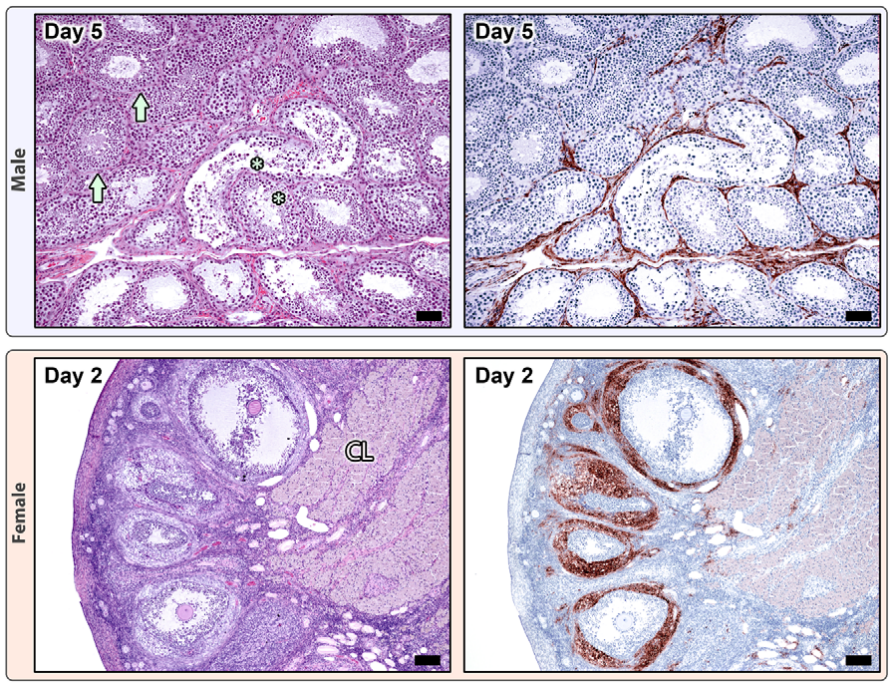
Gonadal changes in males (top) and females (bottom) during smallpox. Males (10^8^ pfu dose), Day 5: Areas of testicular (seminiferous tubule) degeneration (*) adjacent to normal seminiferous tubules (arrows), H&E 10X; mag bar = 50 um. Poxviral IHC staining (brown) is localized to the interstitium surrounding degenerate tubules; normal areas are negative, anti-vaccinia IHC; 10X; mag bar = 50 um. Females (10^9^ pfu), Day 2: H&E shows normal ovary with primordial, primary, secondary, and tertiary follicles, and remnant corpus luteum, H&E 5X; mag bar = 100 um. Poxviral IHC staining is primarily localized to the thecal cell layer of secondary and tertiary follicles, anti-vaccinia IHC; 5X; mag bar = 100 um.

**Figure 5:**
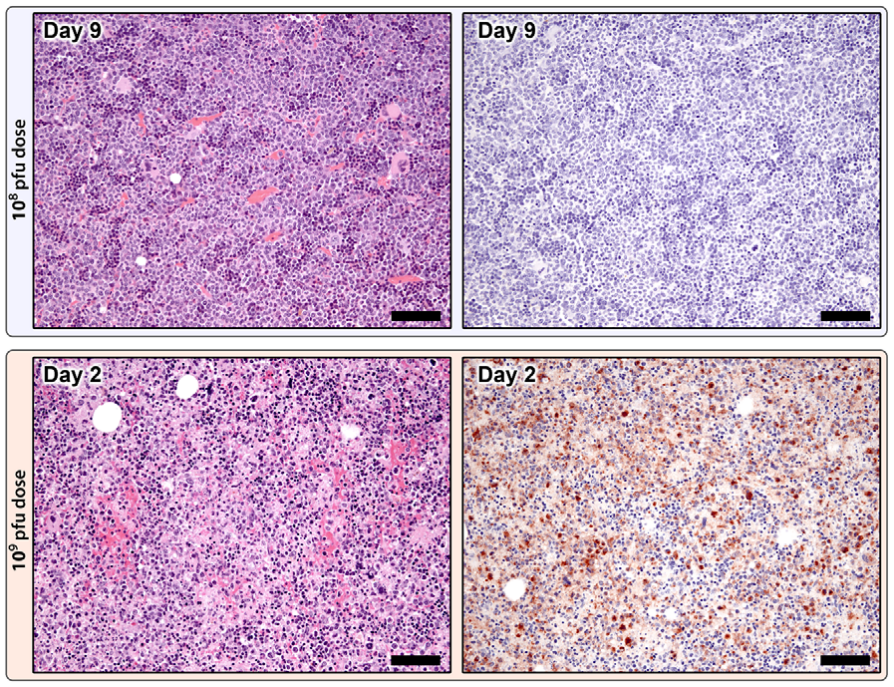
Bone marrow changes in the 10^8^ pfu (top) and 10^9^ pfu (bottom) dose groups. 10^8^ pfu dose, Day 9: Marked myeloid hyperplasia, H&E 20X; mag bar = 50 um. No poxviral IHC staining, anti-vaccinia IHC; 20X; mag bar = 50 um. 10^9^ pfu premature death group, Day 2: Diffuse hematopoietic necrosis and multifocal hemorrhage, H&E 20X; mag bar = 50 um. Widespread poxviral staining (brown), anti-vaccinia IHC; 20X; mag bar = 50 um.

**Figure 6:**
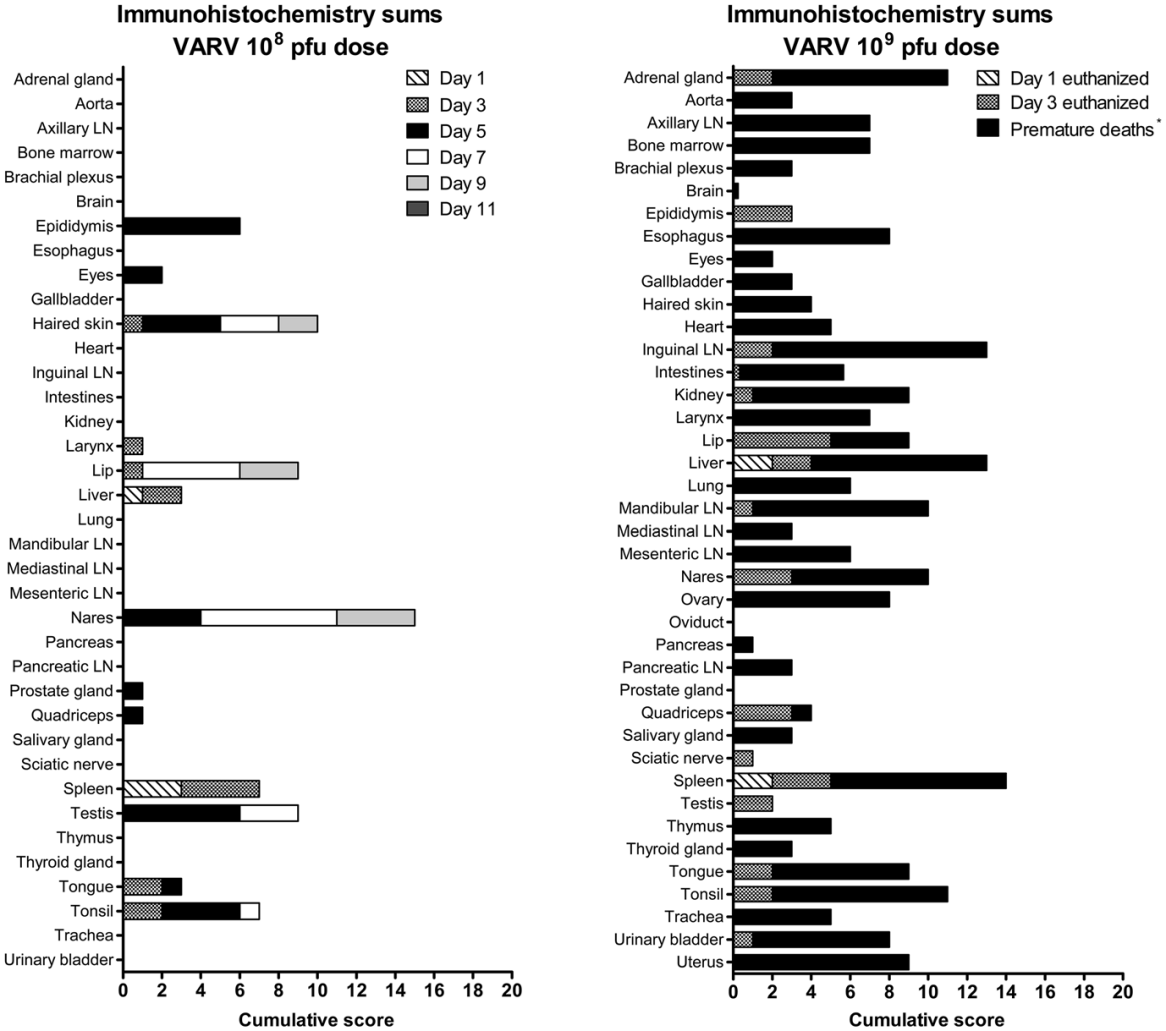
Cumulative poxviral immunohistochemistry scores by tissue type in the 10^8^ pfu and 10^9^ pfu dose groups. IHC stains were subjectively scored on a 0–4 scale (0  =  none; 1 = minimal; 2 = mild; 3 = moderate; 4 = marked). For each tissue type, IHC scores for all 3 animals from the same dose and necropsy day group were summed. If, for an individual animal, more than one slide of the same tissue type was examined and scored, the average score for that tissue type was used in the summation.

Additional histopathologic lesions were found in the testes, bone marrow, and conjunctiva. Testicular degeneration, occasionally accompanied by multiple large foci of lymphoplasmacytic and histiocytic inflammation, first appeared at day 5 and persisted through day 11 (Figure 4). Distinct viral antigen staining was found in the interstitial cells surrounding degenerate seminiferous tubules (Figure 4). Marked myeloid hyperplasia also first appeared on day 5 and persisted through day 11; no viral antigen staining was found within the bone marrow at any time point (Figure 5). One animal in the day 5 group had a minimal neutrophilic conjunctivitis that exhibited positive viral antigen staining. Viral antigen staining by group and tissue type is shown in Figure 6.

#### Histopathology

10^9^ pfu Group.

Despite the lack of gross exanthema or enanthema, typical epithelial hyperplasia, ballooning degeneration and ICIB, as seen in the early stages of the 10^8^ pfu group, were evident histologically at all time points. In the animals euthanized on days 1 and 3, lesions were present in 1/3 and 2/3 of the animals, respectively, and were confined to the oronasal mucosa. Among the animals that were found dead, all exhibited oronasal, cutaneous, and esophageal lesions.

Splenic changes in the animals euthanized on days 1 and 3 resembled those seen in the 10^8^ pfu group, including marginal zone and early germinal center viral antigen staining; however, antigen staining of individual cells within the sinusoids was more common. Splenic changes in the animals that died prematurely drastically differed from all other animals and included marked lymphocytolysis and lymphoid depletion, congestion, neutrophilic inflammation, and small colonies of bacterial cocci (Figure 2D). Viral antigen staining in this group was more robust and distribution also included the periarteriolar lymphoid sheaths (Figure 2E). Ultrastructurally, virions and fibrin were readily apparent (Figure 2F), and poxviral particles were found in the cytoplasm of cells morphologically consistent with macrophages (Figure S1).

Mandibular, mediastinal, axillary, inguinal, and mesenteric lymph nodes were examined, and similar to the 10^8^ pfu group, lymphoid hyperplasia, sinus histiocytosis, and mild hemorrhage, edema, and neutrophilia were present in the animals euthanized on days 1 and 3. Viral antigen staining was limited to scattered macrophages in three lymph nodes. Lymph node changes in the animals that were found dead included marked follicular lymphocytolysis and depletion (Figure 3) and mild to moderate subcapsular sinus hemorrhage. Viral antigen staining was robust and, similar to the spleen, localized to the mantle zone (Figure 3) and macrophages throughout the subcapsular and medullary sinuses. In some nodes, large amounts of antigen were found within depleted germinal centers.

Additional histopathologic lesions were found in the intestinal tract, urinary bladder, bone marrow, gonads, and adrenal glands. Hemorrhage was a significant finding only in the animals that died prematurely and was found in the colon (2/3), small intestine (2/3), urinary bladder (2/3), and iris (1/3). Bone marrow changes were not apparent in the animals euthanized on day 1, and only mild myeloid hyperplasia was seen in 2/3 of the animals euthanized on day 3; no viral antigen staining was seen in either group. In contrast, all of the animals that were found dead exhibited marked hematopoietic necrosis and multifocal hemorrhage with widespread cytoplasmic antigen staining (Figure 5). Ultrastructural findings included hemorrhage, fibrin, and viral particles; Figure S2 demonstrates immature virions within the cytoplasm of both an apoptotic granulocyte and an adjacent lysed cell. Gonadal changes in the animals euthanized on days 1 and 3 were limited to mild testicular degeneration. As in the 10^8^ pfu group, viral antigen staining was localized to interstitial cells adjacent to degenerate tubules. The only female animals in this study were the 3 that died prematurely and no ovarian lesions were found. However, in all 3 animals viral antigen staining was distinct and localized to the thecal cell layer of secondary and tertiary follicles (Figure 4). Mild adrenocortical epithelial degeneration with eosinophilic ICIB was also seen in the animals that died prematurely, and robust viral antigen staining was found primarily in the zona fasciculata. Positive viral antigen staining was also seen in many otherwise normal tissues, as is detailed in Figure 6. Epithelial cells, histiocytes, and vascular pericytes were the most commonly affected cell types; less commonly, endothelial cell staining was noted. The kinetics of viral antigen distribution are discussed below. All three of the animals that died prematurely had histologic evidence of a secondary bacterial infection including acute interstitial pneumonia and/or bacterial hepatitis. Bacteria were evident in several organs including the lung, liver, spleen, and kidney. Gram stain revealed primarily monomorphic colonies of Gram positive cocci, but occasionally small numbers of Gram negative coccobacilli were also seen. The necessary fixation of tissues for removal of samples from biocontainment, unfortunately, made retrospective bacterial cultures and/or PCR impossible. Additionally, because the infections were not clinically apparent, the need for blood or organ cultures was not recognized until after the postmortem exams were complete.

#### Clinical Pathology

Daily hematologic evaluation included total white and red blood cell counts, platelet count, total hemoglobin (Hb), hematocrit (HCT), mean corpuscular volume (MCV), mean corpuscular hemoglobin (MCH), and mean corpuscular hemoglobin concentration (MCHC). Hemogram findings were similar in the both the 10^8^ pfu and 10^9^ pfu groups (Figure S3). Normocytic, normochromic anemia developed at day 3 and progressively worsened to a final mean HCT of 18% on day 11 (−27% change from day 1). Leukocytosis developed in both groups, but occurred earlier in the 10^9^ pfu group than the 10^8^ pfu group (day 2 vs day 5). In the 10^8^ pfu group, total WBC counts peaked at day 6–7 and fell to the upper end of the reference range by day 11. Platelet counts remained within reference range throughout the disease course, indicating that thrombocytopenia did not play a role in the development of hemorrhage in the three 10^9^ pfu dose animals that died prematurely.

Daily serum chemistry evaluation included albumin, total protein, alkaline phosphatase, alanine aminotransferase, aspartate aminotransferase, total bilirubin, blood urea nitrogen, creatinine, cholesterol, glucose, calcium, and amylase. Mild hypoalbuminemia (2–2.5 mg/dL) was the only significant finding and was similar for both the 10^8^ pfu and 10^9^ pfu dose groups (Figure S4). This change in the face of normal total protein and renal and hepatic markers, suggests acute inflammation, increased vascular permeability, or both. Serum globulin concentrations were not measured.

### Variola virus dissemination kinetics

Quantitative rt-PCR and IHC staining were performed to quantify VARV spread *in vivo*. In previous studies viral replication was assessed in all tissues collected by both PCR and plaque assay. Comparison of pfu to genomes showed on average a 200∶1 genome to plaque ratio with no indication of inhibition of PCR in any of the tissues tested. Mean tissue genome concentrations for spleen, liver, bone marrow, lymph node, lung, heart, kidney, testis, brain, and blood are shown for the 10^8^ pfu and 10^9^ pfu dose groups in Figures S5 and S6, respectively. In the 10^8^ pfu dose group, a >1000-fold increase in circulating VARV genome titer occurred over the disease course, and the initial appearance of gross cutaneous lesions correlated with a genome concentration of approximately 10^5^/ml blood (Figure S5). Consistent with iv inoculation and the sinusoidal structure of the spleen and liver, titers in these organs peaked on day 1 (>10^8^/g) and progressively declined to <10^6^/g by day 11. The highest titers, in excess of 10^9^genomes/g, occurred in bone marrow on days 3 and 5 and in testis on day 5. Indeed, testis had the highest genome concentrations of all organs examined and reached a peak titer of 5.6×10^9^ genomes/g on day 5 post-exposure. Lymph node titers remained stable at 10^6^–10^7^/g throughout the course of the study.

Genome titers in the 10^9^ pfu group were greater than those in the 10^8^ pfu group (Figures S5 & S6). Among the animals that received the 10^9^ pfu dose, viral genome titers in all tissues examined were greatest in the animals that died prematurely. The highest concentrations were observed in the spleen and exceeded 10^13^/g tissue. Similar to the 10^8^ pfu group, liver, bone marrow and spleen were primary targets of infection.

Consistent with the qrt-PCR findings, VARV antigen was detected immunohistochemically as early as day 1 post-exposure in the spleen and liver in both groups (Figure 6). In the 10^8^ pfu group, at 3 days post-infection antigen persisted in the spleen and liver and also became apparent in haired skin, oral and laryngeal mucosa, and tonsil. By mid-infection (days 5–7), splenic and hepatic involvement was largely cleared, epidermal and mucosal epithelial involvement persisted, and the sex organs (prostate, epididymis, and testis) became involved. At later time points (days 9–11), VARV antigen was predominantly detected in the skin and oronasal mucosa.

In the 10^9^ pfu group, antigen distribution varied by group. Findings in the animals euthanized on days 1 and 3 were similar to those seen in the 10^8^ pfu group except that distribution on day 3 extended beyond the spleen, liver, skin, and mucosa to include urogenital organs, occasional lymph nodes, colon, and adrenal gland. In contrast, spread of virus in the animals that died prematurely was explosive with robust staining found in most tissues regardless of whether they died on day 2 or 3 post exposure. As noted above, epithelial cells, histiocytes, and vascular pericytes were the most commonly infected cell types. Skeletal muscle staining at the injection site was seen in several animals.

## Discussion

We previously described a cynomolgus macaque model for smallpox. In the present study, this model was refined with a focus on the temporal effects of VARV on the host. Special emphasis was placed on the virological, immunological, and pathological changes over time in both ordinary and hemorrhagic models of smallpox. Understanding the progression of disease will help to identify potential time points for therapeutic intervention, as well as fulfill requirements of the US Food and Drug Administration (FDA) Animal Efficacy Rule established to facilitate the acceptance of animal models for drug efficacy testing.

In the present study, two clinical types of variola major disease were modeled: ordinary and hemorrhagic. Similar to human infections, these two forms differed in their clinical presentation, time to death, and overall severity of disease. Interestingly, inoculation dose was not the sole determinant of disease manifestation, as not all animals in the 10^9^ pfu group developed hemorrhagic smallpox. This was unexpected since our previous studies showed a strong correlation between high inoculation dose and the occurrence of hemorrhagic disease [15]. In this study, hemorrhagic disease developed only in animals that also acquired a secondary systemic bacterial infection, suggesting the bacterial infection potentiated the viral infection. This was most strikingly reflected in the more widespread and robust viral antigen staining in the animals that developed hemorrhagic disease. While initially alarming, this observation corroborates the fidelity of the primate model in recapitulating the lethal form of human smallpox. Historically, systemic bacterial infections were especially common in cases of hemorrhagic smallpox and much speculation centered on the role of these organisms in smallpox pathogenesis [12]. This finding has also been observed in experimental monkeypox virus infections in cynomolgus macaques where despite receiving equivalent doses, hemorrhagic disease only occurred when a concurrent secondary bacterial infection was present [16]. These findings suggest concurrent infections are an important variable in the differential development of hemorrhagic smallpox over ordinary smallpox.

Another important difference unique to the animals that developed hemorrhagic smallpox is sex. Only three female animals were included in this study and all three developed hemorrhagic-like smallpox and died. Cell mediated and humoral immune responses differ among males and females, and in females the response varies according to menstrual cycle stage [17], [18]. Given that Th2 immune responses predominate during the luteal phase (including pregnancy) [19], [20], and Th1 responses are responsible for anti-viral immunity [21], [22], [23], it is possible that sex steroid hormone-associated effects on the immune system may have influenced VARV pathogenesis. The historical literature supports this possibility as it is widely documented that hemorrhagic smallpox was more common in pregnant women even in the face of previous variolation or vaccination [24].

Despite having relatively few animals develop hemorrhagic smallpox, we observed several key differences between the ordinary and hemorrhagic forms. Most notably, distinct morphologic differences in the lymphoid and hematopoietic responses to VARV infection were found. In the ordinary model, lymphoid and myeloid hyperplasias were consistently present, whereas marked lymphocytolysis and hematopoietic necrosis were found in the hemorrhagic model. Viral antigen staining patterns also differed. In the ordinary model, only low level staining was found in what appear to be marginal zone macrophages and/or dendritic cells on days 1 and 3 post exposure, coincident with the onset of lymphoid hyperplasia. Beyond that no viral antigen was found and lymphoid hyperplasia persisted, suggesting effective viral clearance and an ongoing active immune response. Similarly, the nodal and splenic plasmacytosis seen at later time points suggests effective development of the adaptive immune response. It is interesting that while viral antigen staining was found in the spleen (and not the lymph nodes), significantly more robust and persistent lymphoid hyperplasia occurred in the lymph nodes, perhaps indicating that while primary clearance and processing of the blood-borne antigen occurs in the spleen, trafficking to the nodal lymphocyte population is important for development of an effective adaptive response.

In contrast to the ordinary model, viral antigen staining in the animals that developed hemorrhagic disease was widespread and involved many tissue types including the lymph nodes and bone marrow. Within the spleen, antigen staining was not limited to the marginal zone; periarteriolar lymphoid sheaths and large numbers of sinusoidal macrophages were also involved. In addition, in both the spleen and lymph nodes the depleted germinal centers often contained abundant apoptotic bodies mixed with viral antigen, suggesting exhaustion of the adaptive response and incomplete viral clearance. This may explain the consistently higher viral titers in both blood and tissues, and the more widespread viral antigen staining seen in these animals. Similar to what has been shown for other orthopoxvirus infections [25], in both the ordinary and hemorrhagic forms we confirmed macrophage infection via positive viral antigen staining and presence of virus ultrastructurally. Thus, it is possible that with an incomplete or ineffective adaptive response, whether due to high viral load, concurrent infections, or physiologic state, viral trafficking within macrophages proceeds unchecked. It is also noteworthy that in their review of human smallpox, Councilman *et al* describe lymphoid and hematopoietic necrosis in the hemorrhagic cases only, a finding which lends validity to the macaque model of hemorrhagic smallpox [12].

In the hemorrhagic model, expanded distribution of viral antigen involved not only additional tissue types, but also additional cell types. Orthopoxviruses are widely known to be epitheliotropic and we found that to be true in both our ordinary and hemorrhagic models. Epithelial cells in haired skin and mucosa were a primary target in both models, and in the hemorrhagic model, hepatocytes, pneumocytes, adrenocortical epithelium, and urogenital epithelium were also commonly involved. Likewise, as discussed above, macrophages were found to be infected in both models, with more widespread involvement in the hemorrhagic model. Interestingly, we also found positively stained pericytes, and to a lesser extent endothelial cells, in tissues from the animals with hemorrhagic smallpox. In fact, for many of the additionally affected tissues in the hemorrhagic model, pericytes were the primary positive cell type. Similar findings have been reported for cynomolgus macaques experimentally infected with monkeypox virus [16]. Given the role of pericytes in maintaining the integrity of small capillaries and post-capillary venules, it is possible that viral-associated dysfunction of these cell types may be a primary mechanism by which hemorrhage develops. Less commonly, endothelial cells were found to be involved, thus they may also contribute to the loss of vascular wall integrity. Other possible mechanisms for hemorrhage include thrombocytopenia and coagulation cascade disorders. Thrombocytopenia did not occur in either model, and coagulation factors were not evaluated. This is a critical area in need of further investigation.

We also noted several similarities between the ordinary and hemorrhagic disease forms. Clinicopathologically, normocytic normochromic anemia developed in both groups. While hemorrhage may have played a role in the animals that developed hemorrhagic smallpox, this was not the responsible mechanism in the animals with ordinary smallpox. Other possible mechanisms include impaired erythrocyte production or hemolysis. The hematopoietic necrosis observed in the hemorrhagic smallpox model involved all three cell lineages, including the erythroid population. However, given the short (2–3 days) disease course, it is not likely that this played a major role. Hyperbilirubinemia was not detected in either group, indicating that if hemolysis occurred, it was not of sufficient magnitude to exceed the capacity of the liver to remove bilirubin. Additional findings to support or refute a hemolytic mechanism, such as serum phosphorous concentrations and erythrocyte cytology were not performed. Another possibility is ‘anemia of inflammatory disease’ which, despite its alternate moniker ‘anemia of chronic disease’, can manifest in as little as 3–10 days. In both smallpox models, anemia first developed at day 3, and in the ordinary model, it persisted through day 11. Thus, this may be the mechanism responsible for the anemia; however, further characterization is warranted. Progressive hypoalbuminemia was also common to both groups. As with the anemia, hemorrhage likely played a role in the animals that developed hemorrhagic smallpox, but not in the ordinary smallpox model. Renal, intestinal, or hepatic pathology sufficient to result in protein-losing nephropathy/enteropathy or reduced hepatic albumin production was not found. In addition, clinical pathology data supports normal renal and hepatic function. Given that albumin is a negative acute phase protein, it is likely that acute inflammation is the primary mechanism responsible for the reduced serum concentrations in the ordinary smallpox group.

Compared to what is known about human smallpox, our models resemble the human condition in time to disease onset with hemorrhagic smallpox with respect to viremia, and gross and histopathologic appearance of most lesions. Key areas in which our macaque models differ from the human disease include the lack of thrombocytopenia in macaques with hemorrhagic smallpox, and the development of significant lymphadenopathy in macaques with ordinary smallpox. In humans, thrombocytopenia is documented in cases of hemorrhagic smallpox and lymphadenopathy is not a prominent finding in either form of the disease. The reason for these differences is not clear. In ordinary smallpox in humans, Councilman *et al* describe edema and sinus histiocytosis as the primary pathologic change in lymph nodes, and they specifically state that the enlargement is “due more to edema than cellular hyperplasia” [12]. In contrast, they describe consistent lymphoid hyperplasia in the spleen [12]. In the macaque ordinary smallpox model, lymphoid hyperplasia is minimal in the spleen and marked in the lymph node, with lymph node edema being only a minor change.

**Figure 7:**
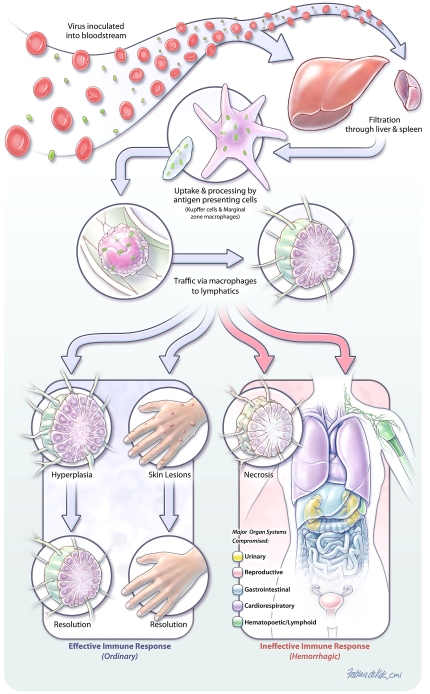
Model of VARV pathogenesis. Viremic blood is filtered through the spleen and liver following inoculation. Within 24 hours marginal zone macrophages and Kupffer cells begin expressing viral antigen indicating likely uptake and processing of the pathogen. When the immune response is effective these antigen presenting cells then traffic via the lymphatics to the lymph nodes where a hyperplastic response develops. Concurrently, trafficking to other preferred sites such as skin occurs, and lesions progress and regress as the ongoing adaptive immune response controls and clears the pathogen. In the face of an ineffective immune response, trafficking via macrophages also occurs, however, lymphoid necrosis rather than hyperplasia occurs, allowing unabated trafficking of the virus to other tissues. Infected cells undergo necrosis, pyroptosis, and/or apoptosis which, given the widespread state of infection, results in inflammation, hemorrhage and death.

Based on the temporal progression of virologic, pathologic, and immunologic events after experimental inoculation with variola virus, our findings suggests that the pathogenesis diverges at the initial viral clearance stage to produce either the ordinary or hemorrhagic form of the disease (Figure 7). In both intravenous inoculation models, viremic blood is filtered through the spleen and liver. Within 24 hours marginal zone macrophages and Kupffer cells begin expressing viral antigen indicating likely uptake and processing of the pathogen. When the immune response is effective these antigen presenting cells then traffic via the lymphatics to the lymph nodes where a hyperplastic response develops. Concurrently, trafficking to other preferred sites such as skin and gonads occurs, and lesions progress and regress as the ongoing adaptive immune response controls and clears the pathogen. In the face of an ineffective immune response, trafficking via macrophages also occurs. However, lymphoid necrosis rather than hyperplasia occurs, allowing unabated trafficking of the virus to other tissues. As in ordinary smallpox, skin and gonads are targeted, but, in this situation epithelial and steroidogenic cells in other organs, such liver, lung, kidney, and adrenal gland are infected. In addition, we see antigen expression in cells that are not normally regarded as preferred poxviral targets, such as endothelial cells, pericytes, and hematopoietic cells. The reason for this additional distribution is not clear. It may be a reflection of viral load, where the lack of an effective immune response allows production of large numbers of infectious virions which then go on to infect the additional cell types. The infected cells then undergo necrosis, apoptosis, and/or pyroptosis which, given the widespread state of infection, results in inflammation, hemorrhage and death.

This study has expanded our knowledge of both the ordinary and hemorrhagic forms of smallpox, however, many areas warrant further investigation. As discussed above, determining the molecular mechanisms responsible for cellular tropism and effective innate and adaptive immune responses is crucial for identifying targets of therapeutic intervention. In addition, the mechanisms of cell death in VARV infection are unclear and elucidation of these pathways may reveal additional therapeutic avenues. Further characterization of the reproductive pathology is also justified. In both males and females, gonads are a primary target of many orthopoxviruses, and there is a high risk of spontaneous abortion among pregnant women infected with variola virus. While direct viral infection of the fetus has been documented, it is not common, and more often the abortus presents without pathologic evidence of poxviral infection. Our IHC findings suggest that viral targeting of thecal cells and subsequent reduced progesterone production may be one mechanism by which fetal loss occurs. In addition, historically, regardless of vaccination or variolation status, pregnant women were at significantly increased risk for developing fatal infections when infected with VARV [24]. Given the unique reproductive physiology of human and non-human Old World primates, the macaque model may be useful for determining the reasons behind this enhanced susceptibility. Finally, the appropriateness of any animal model is based on its resemblance or non-resemblance to the human condition. In the case of VARV, historical reports allude to histopathologic variations in the human condition. In addition to the disparate lymphoid responses discussed above, Councilman *et al* also reported that syncytia are common in human smallpox [12], a salient feature which is lacking in the macaque models. Given the importance of developing valid and reliable animal models to satisfy the FDA Animal Efficacy Rule, it would be wise to apply, to the extent possible, modern techniques to archived human smallpox tissue in order to have a valid comparator for development and refinement of animal models.

## Materials and Methods

### Ethics statement

This study was carried out in strict accordance with the recommendations in the Guide for the Care and Use of Laboratory Animals of the National Institutes of Health. The protocol was approved by the Institutional Animal Care and Use Committee at the Centers for Disease Control and Prevention, (Protocol JAHMONC1400). All research involving non-human primates was performed in accordance with the recommendations of the Weatherall report, "The use of non-human primates in research". Animal were anesthetized intramuscularly (im) with tiletamine-zolazepam (3 mg/kg), and all efforts were made to minimize suffering.

### Animals and virus

Twenty-seven, vaccinia virus-naïve cynomolgus macaques *(Macaca fascicularis)* were anesthetized intramuscularly (im) with tiletamine-zolazepam (3 mg/kg), and all efforts were made to minimize suffering. Anesthetized animals were injected intravenously (iv) with either 10^8^ (n = 18) or 10^9^ (n = 9) pfu of VARV Harper strain. The virus seed was prepared as previously described. Animals were examined at least twice daily post-exposure and sedated with tiletamine-zolazepam prior to all phlebotomy and euthanasia procedures. Animals in the 10^8^ pfu group were scheduled for euthanasia and necropsy at days 1, 3, 5, 7, 9 and 11 post infection; animals in the 10^9^ pfu group were scheduled at days 1, 3 and 4. The study design and outcome of individual animals is listed in Table S1. All work involving nonhuman primates (NHPs) infected with VARV was performed at the Maximum Containment Laboratory, Centers for Disease Control and Prevention, in Atlanta, Georgia, USA. All animals were handled in accord with the *Guide for the Care and Use of Laboratory Animals*, and the Institutional Animal Care and Use Committee at the Centers for Disease Control and Prevention.

### Enzyme-linked immunosorbent assay (ELISA)

Cytokine/chemokine concentrations in NHP sera/plasma were assayed using commercially available ELISA kits according to the manufacturers' directions. Cytokines/chemokines assayed included NHP interleukin (IL)-2, IL-4, IL-10, IL-12, interferon (IFN)- γ, and tumor necrosis factor (TNF)- α (BioSource International, Inc., Camarillo, CA). ELISAs for the detection of human proteins known to be compatible with cynomolgus macaques included IL-6, IFN-α, IFN-β, macrophage inflammatory protein 1 alpha (MIP-1α), and MIP-1β (BioSource), and human IL-1β, IL-8, IL-18, and monocyte chemotactic protein (MCP)-1 (R&D Systems, Minneapolis, MN).

### Quantitative real-time PCR

qrt-PCR was performed as previously described [26]. Briefly, tissues were sectioned into media to generate a 10% homogenate. The homogenate was subsequently freeze-thawed and sonicated 4 times, centrifuged, and clarified supernatant was removed for extraction. DNA was extracted from ethylenediaminetetraacetic acid (EDTA) blood to analyze virus load. All samples were lysed and extracted using the QIAamp Mini Kit (QIAGEN, Valencia, CA) according to the manufacturer's instructions. qrt-PCR was performed with the LightCycler (Roche Applied Science, Indianapolis, IN) by use of a pan-Orthopoxvirus assay as described elsewhere [26] to determine genomes per mg or ml of tissue.

### Histopathology and Immunohistochemistry

All NHP postmortem exams were performed on the day of death. The following tissues were collected at necropsy: normal skin, mammary gland, skin with rash and/or pox lesions, inguinal, axillary, mandibular, mediastinal, and mesenteric lymph nodes, salivary gland, testis or ovary, liver, gallbladder, spleen, adrenal gland, left and right kidneys, lung, heart, tongue, tonsil, larynx, thyroid gland, parathyroid gland, trachea, esophagus, urinary bladder, prostate gland or uterus, stomach, pancreas, duodenum, jejunum, ileum, cecum with ileocecal junction, colon, nares, upper lip, eyes, brain, pituitary gland, sciatic nerve, skeletal muscle, and femoral bone marrow. Tissues were fixed in 10% neutral buffered formalin, embedded in paraffin, sectioned at 5–6 µm, and stained with hematoxylin & eosin (H&E) according to established protocols [27].

VARV antigen was identified immunohistochemically using rabbit anti-vaccinia virus polyclonal antibody (1∶5000; Antibody #1069, USAMRIID, Frederick, MD) incubated for 30 minutes at room temperature (RT). Primary antibody was localized with anti-rabbit biotinylated secondary antibody (30 minutes at RT), horseradish peroxidase and diaminobenzidine substrate. Sections were counterstained with hematoxylin followed by Scott's tap water, and examined by light microscopy by board-certified veterinary pathologists (JAC, AJJ, and JLR). IHC stains were subjectively scored on a 0–4 scale (0 = none; 1 = minimal; 2 = mild; 3 = moderate; 4 = marked). For each tissue type, IHC scores for all 3 animals from the same dose and necropsy day group were summed. If, for an individual animal, more than one slide of the same tissue type was examined and scored, the average score for that tissue type was used in the summation.

### Electron Microscopy

Skin, inguinal lymph node, liver, spleen, lung, heart, and bone marrow were analyzed by transmission electron microscopy (TEM). Tissues were fixed in 2.5% glutaraldehyde in 0.1 M phosphate buffer (pH 7.3), irradiated (5×10^6^ rads), and processed as described [28]. Ultrathin sections were cut, placed on 200-mesh copper electron microscopy grids, stained with uranyl acetate and lead citrate. Ultrathin sections were examined at 80 kV on a JOEL 1011 transmission electron microscope.

## Supporting Information

Figure S1 **10^9^ pfu premature death group.** Ultrastructural appearance of immature poxvirus virions within the cytoplasm of a hemosiderin (*) laden macrophage, TEM; 10000X; mag bar = 2 um.
(TIF)

Figure S2 **10^9^ pfu premature death group.** Ultrastructural appearance of immature poxvirus virions (thick arrow) within the cytoplasm of a both an apoptotic granulocyte and an adjacent lysed cell (thin arrow), TEM; 12000X; mag bar = 2 um.
(TIF)

Figure S3 **Hemogram findings in the 10^8^ pfu (blue) and 10^9^ pfu (red) dose groups.** Gray areas indicate the normal reference range. Error bars indicate standard error of the mean (SEM).
(TIF)

Figure S4 **Clinical chemistry findings in the 10^8^ pfu (blue) and 10^9^ pfu (red) dose groups.** Gray areas indicate the normal reference range.
(TIF)

Figure S5 **Mean tissue genome concentrations in the 10^8^ pfu group.**
(TIF)

Figure S6 **Mean tissue genome concentrations in the 10^9^ pfu dose group.**
(TIF)

Table S1 **Study Design.**
(DOCX)
