# Supplementary figures and images for: Progression of Pathogenic Events in Cynomolgus Macaques Infected with Variola Virus
Source: PLoS One. 2011 Oct 6;6(10):e24832. doi: 10.1371/journal.pone.0024832 (PMC3188545; doi:10.1371/journal.pone.0024832)

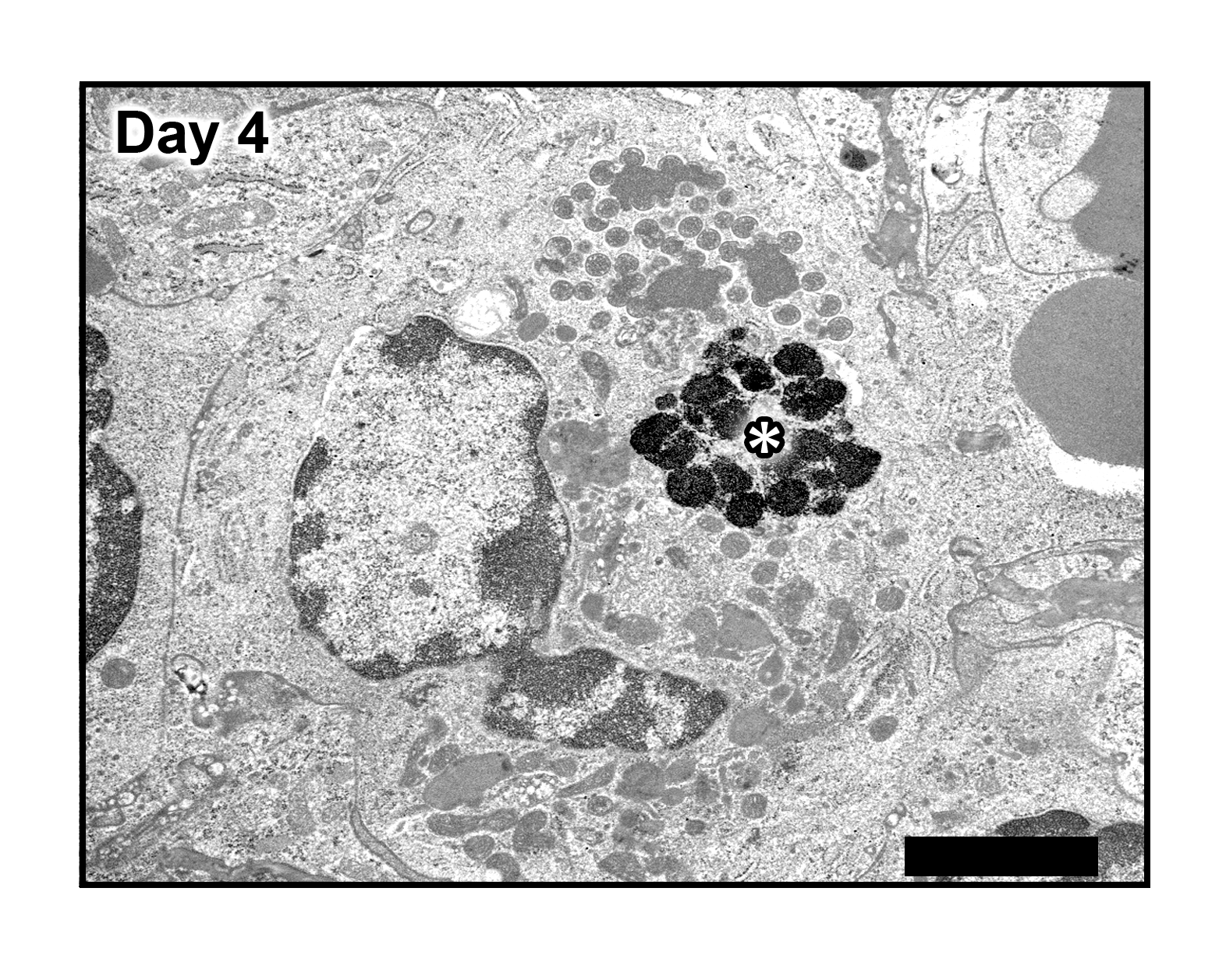

Supplement: Figure S1 — 109 pfu premature death group. Ultrastructural appearance of immature poxvirus virions within the cytoplasm of a hemosiderin (*) laden macrophage, TEM; 10000X; mag bar = 2 um. (TIF) [file pone.0024832.s001.tif]

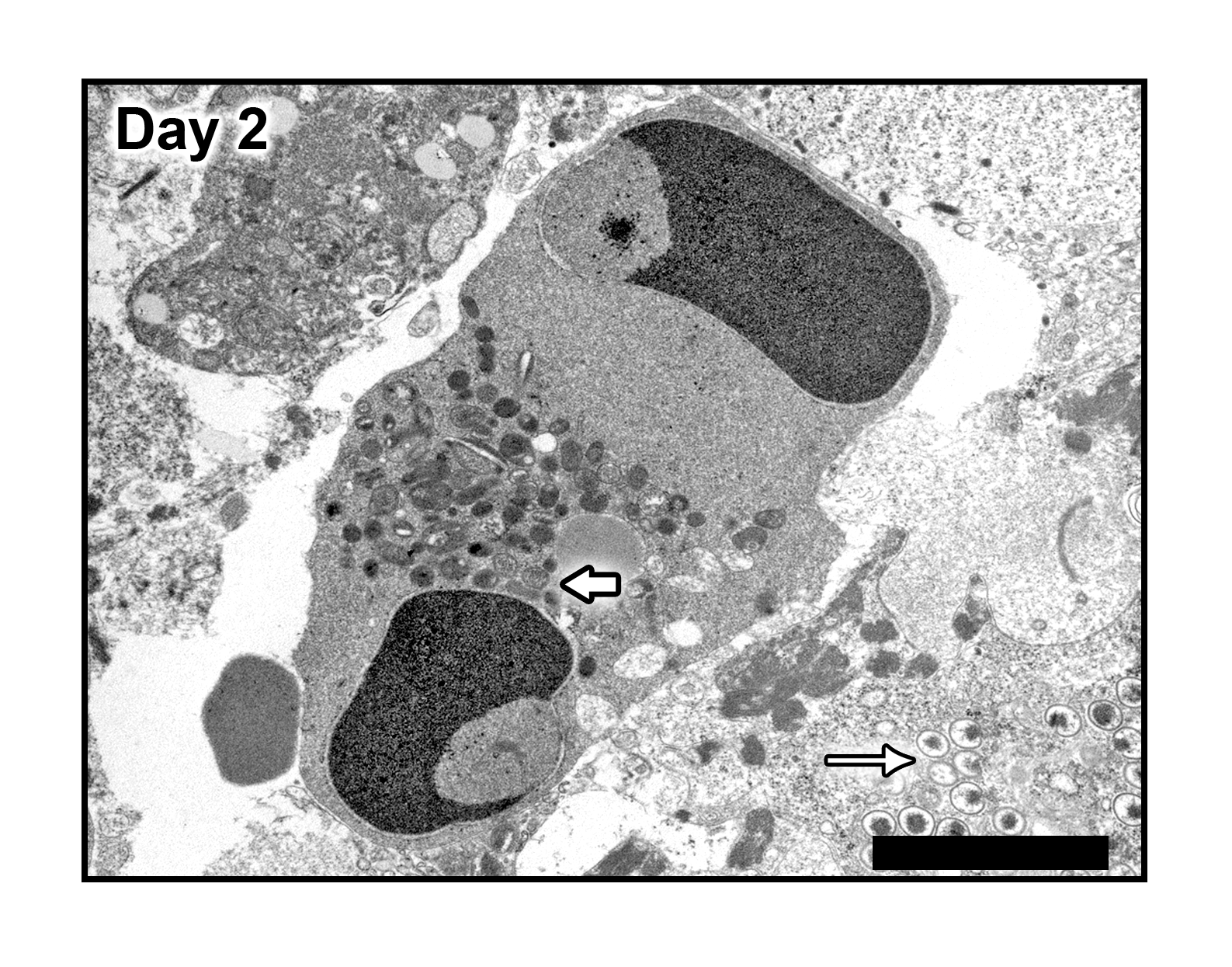

Supplement: Figure S2 — 109 pfu premature death group. Ultrastructural appearance of immature poxvirus virions (thick arrow) within the cytoplasm of a both an apoptotic granulocyte and an adjacent lysed cell (thin arrow), TEM; 12000X; mag bar = 2 um. (TIF) [file pone.0024832.s002.tif]

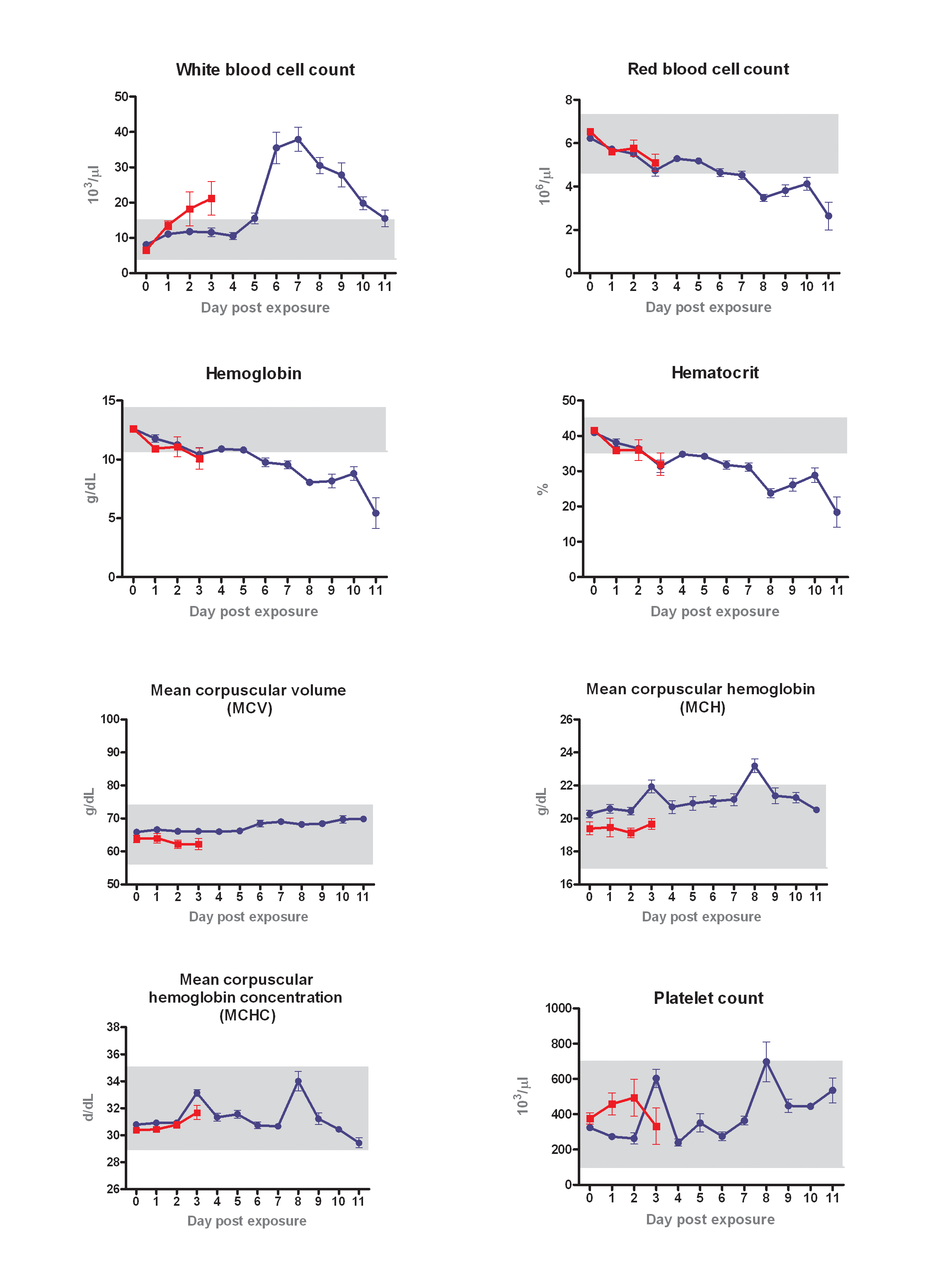

Supplement: Figure S3 — Hemogram findings in the 108 pfu (blue) and 109 pfu (red) dose groups. Gray areas indicate the normal reference range. Error bars indicate standard error of the mean (SEM). (TIF) [file pone.0024832.s003.tif]

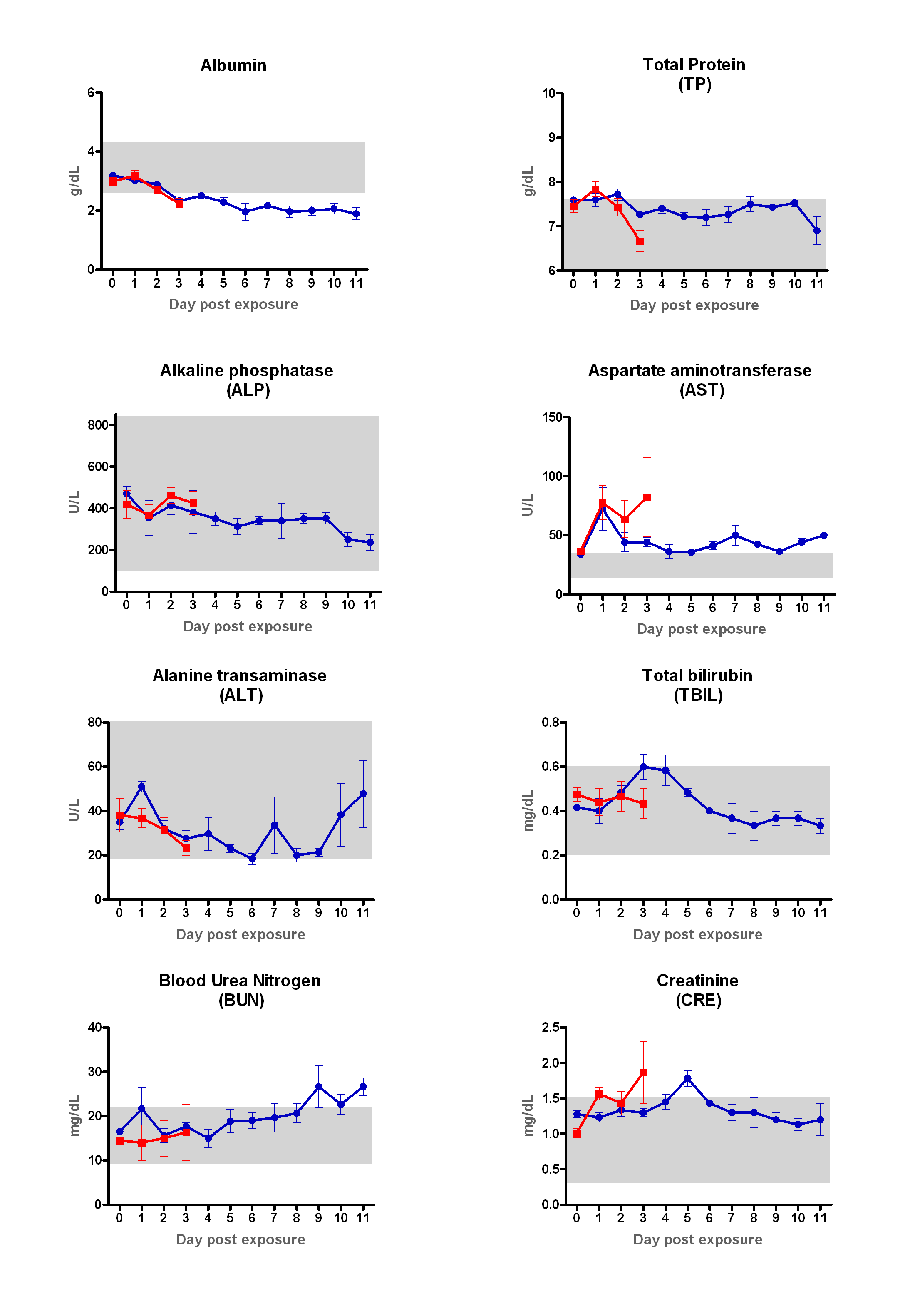

Supplement: Figure S4 — Clinical chemistry findings in the 108 pfu (blue) and 109 pfu (red) dose groups. Gray areas indicate the normal reference range. (TIF) [file pone.0024832.s004.tif]

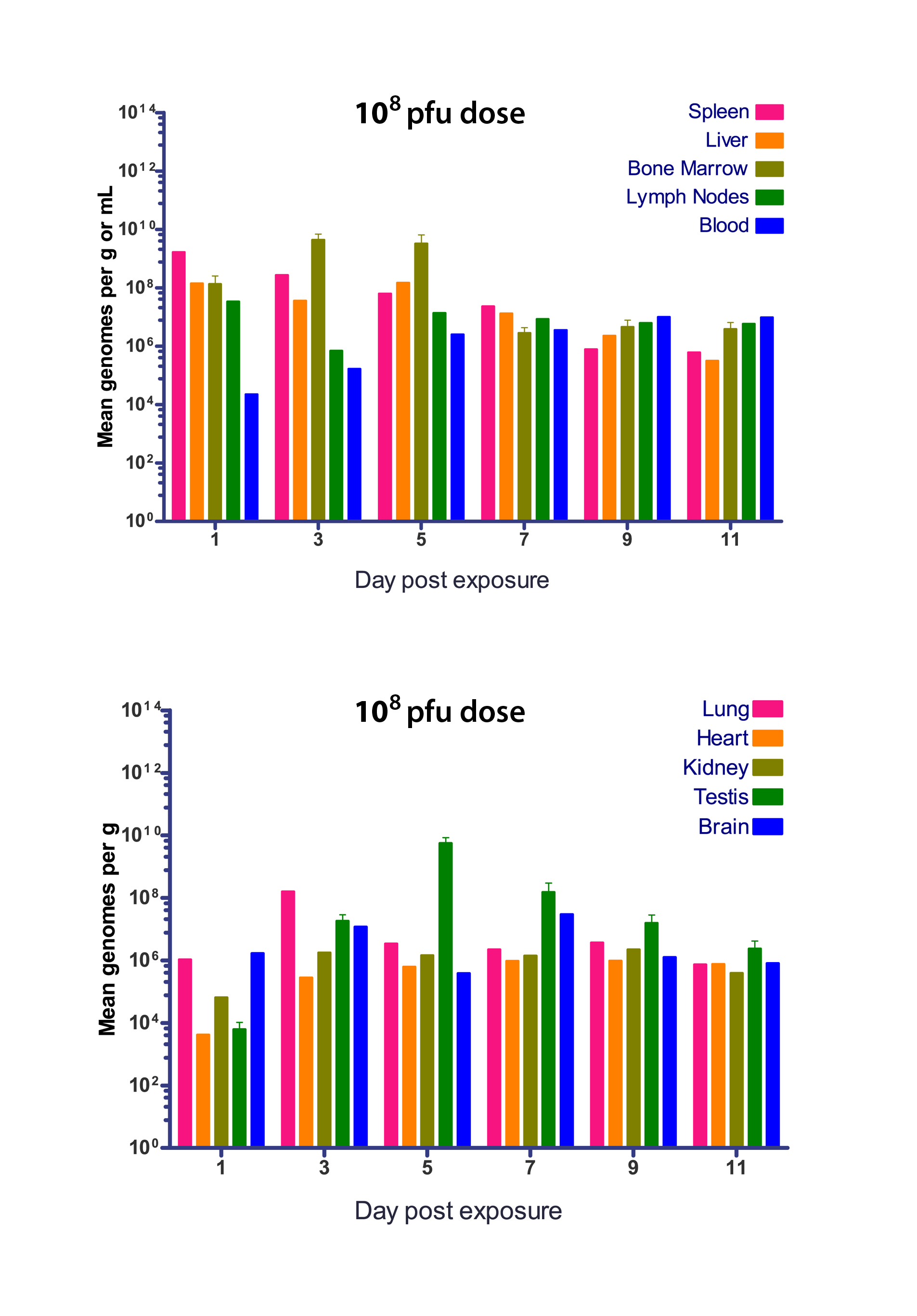

Supplement: Figure S5 — Mean tissue genome concentrations in the 108 pfu group. (TIF) [file pone.0024832.s005.tif]

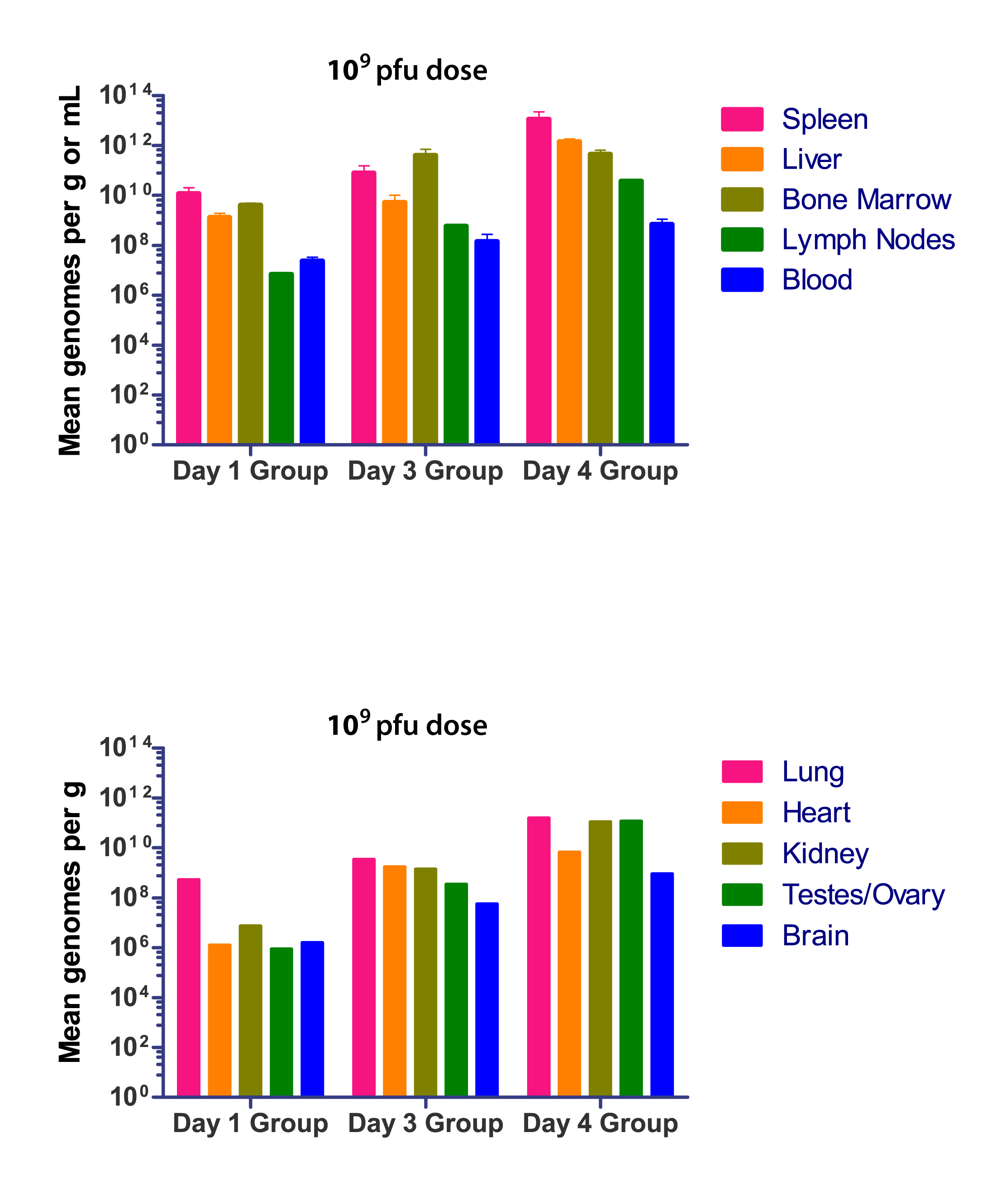

Supplement: Figure S6 — Mean tissue genome concentrations in the 109 pfu dose group. (TIF) [file pone.0024832.s006.tif]
