# Supplementary material for: Progression of Pathogenic Events in Cynomolgus Macaques Infected with Variola Virus
Source: PLoS One. 2011 Oct 6;6(10):e24832. doi: 10.1371/journal.pone.0024832 (PMC3188545; doi:10.1371/journal.pone.0024832)
Supplement: Table S1 — Study Design. (DOCX) [file pone.0024832.s007.docx]

**Supplemental Table 1. Study Design**

| **Group (Virus Dose)** | **NHP ID #** | **Gender** | **Day** |
| --- | --- | --- | --- |
| **10^8^ pfu** | 8892 | M | 1 |
|  | 8881 | M | 1 |
|  | 9191 | M | 1 |
|  | 9011 | M | 3 |
|  | 9444 | M | 3 |
|  | 9061 | M | 3 |
|  | 9296 | M | 5 |
|  | 9064 | M | 5 |
|  | 8886 | M | 5 |
|  | 9391 | M | 7 |
|  | 8879 | M | 7 |
|  | 9155 | M | 7 |
|  | 9281 | M | 9 |
|  | 9387 | M | 9 |
|  | 9034 | M | 9 |
|  | 9600 | M | 11 |
|  | 9438 | M | 11 |
|  | 9425 | M | 11 |
|  |  |  |  |
| **10^9^ pfu** | 9140 | M | 1 |
|  | 9440 | M | 1 |
|  | 9141 | M | 1 |
|  | 8992 | M | 3 |
|  | 9180 | M | 3 |
|  | 9143 | M | 3 |
|  | 9808 | F | 4 (2)^*^ |
|  | 9801 | F | 4 (3)^*^ |
|  | 9805 | F | 4 (3)^*^ |

^*^Animals were scheduled for necropsy on day 4 but died at an earlier point in time. The actual number of days post-exposure is shown in parentheses. Animals 9808, 9801, and 9805 were the only ones to display hemorrhagic disease. NHP, nonhuman primate; M, male; F, female; pfu, plaque-forming units.
